# Region and Cell Type Distribution of TCF4 in the Postnatal Mouse Brain

**DOI:** 10.3389/fnana.2020.00042

**Published:** 2020-07-17

**Authors:** Hyojin Kim, Noah C. Berens, Nicole E. Ochandarena, Benjamin D. Philpot

**Affiliations:** ^1^Department of Cell Biology & Physiology, University of North Carolina at Chapel Hill, Chapel Hill, NC, United States; ^2^Neuroscience Center, University of North Carolina at Chapel Hill, Chapel Hill, NC, United States; ^3^Department of Biology, University of North Carolina at Chapel Hill, Chapel Hill, NC, United States; ^4^MD-Ph.D. Program, University of North Carolina at Chapel Hill, Chapel Hill, NC, United States; ^5^Carolina Institute for Developmental Disabilities, University of North Carolina at Chapel Hill, Chapel Hill, NC, United States

**Keywords:** transcription factor 4, Pitt-Hopkins syndrome, schizophrenia, autism spectrum disorder, neurodevelopmental disorder, intellectual disability

## Abstract

Transcription factor 4 is a class I basic helix-loop-helix transcription factor regulating gene expression. Altered *TCF4* gene expression has been linked to non-syndromic intellectual disability, schizophrenia, and a severe neurodevelopmental disorder known as Pitt-Hopkins syndrome. An understanding of the cell types expressing TCF4 protein in the mouse brain is needed to help identify potential pathophysiological mechanisms and targets for therapeutic delivery in TCF4-linked disorders. Here we developed a novel green fluorescent protein reporter mouse to visualize TCF4-expressing cells throughout the brain. Using this TCF4 reporter mouse, we observed prominent expression of TCF4 in the pallial region and cerebellum of the postnatal brain. At the cellular level, both glutamatergic and GABAergic neurons express TCF4 in the cortex and hippocampus, while only a subset of GABAergic interneurons express TCF4 in the striatum. Among glial cell groups, TCF4 is present in astrocytes and immature and mature oligodendrocytes. In the cerebellum, cells in the granule and molecular layer express TCF4. Our findings greatly extend our knowledge of the spatiotemporal and cell type-specific expression patterns of TCF4 in the brain, and hence, lay the groundwork to better understand TCF4-linked neurological disorders. Any effort to restore TCF4 functions through small molecule or genetic therapies should target these brain regions and cell groups to best recapitulate TCF4 expression patterns.

## Introduction

Transcription factor 4 (TCF4, Gene ID 6925) is a basic helix-loop-helix (bHLH) transcription factor, acting as both a repressor and activator of gene expression (Massari and Murre, 2000). The protein’s functional domains include a first activation domain, a nuclear localization signal, a second activation domain, and a bHLH domain. The bHLH domain consists of a basic region that directly mediates DNA binding and amphipathic helices that provide a dimerization interface. TCF4 can form homo- and hetero-dimers with cell type-specific bHLH proteins, which modulate its function (Murre et al., 1994). The human TCF4 gene can be transcribed from multiple promoters, and the usage of alternative 5′ exons and splicing produces protein isoforms with 18 different N′-termini and variable functional domains (Sepp et al., 2011). Genomic alterations that affect TCF4 function or levels increase the risk of neurodevelopmental or psychiatric disorders (Sepp et al., 2012; Bedeschi et al., 2017). For example, haploinsufficiency of *TCF4* is the main pathogenic mechanism in Pitt-Hopkins syndrome (PTHS), which is characterized by intellectual disability, sensory processing deficits, anxiety, and speech and motor delay (Amiel et al., 2007; Zweier et al., 2007). PTHS is associated with enlarged ventricles, cerebellar atrophy, and hippocampal and corpus callosum hypoplasia (Peippo et al., 2006; Amiel et al., 2007; Zweier et al., 2008; Goodspeed et al., 2018; Zollino et al., 2019), suggesting that gross brain development is sensitive to dramatic changes in *TCF4* expression and function. More subtle alterations in *TCF4* gene expression have been linked to non-syndromic intellectual disability, schizophrenia, and bipolar diseases (Pickard et al., 2005; Kharbanda et al., 2016; Maduro et al., 2016; Forrest et al., 2018; Ma et al., 2018; Mary et al., 2018). These structural and behavioral phenotypes emphasize the importance of *TCF4* gene regulation for normal brain function.

Mouse models carrying mutations or deletions of the bHLH region of *Tcf4* display many PTHS-like phenotypes, including memory and learning deficits, anxiety, hyperactivity, and sensory dysfunction. Perturbations of *Tcf4* disrupt synaptic function in the hippocampus and cortex, likely contributing to impaired learning and memory (Kennedy et al., 2016; Rannals et al., 2016; Thaxton et al., 2018). At the cellular level, reduced TCF4 protein levels impair dendritic development, neuronal migration, and cortical laminar organization (Chen et al., 2016; Li et al., 2019; Wang et al., 2020). In glial cells, TCF4 loss leads to delayed differentiation of oligodendrocyte progenitors (Fu et al., 2009). Thus, evidence from mouse studies implicates TCF4 in a variety of critical processes in brain development and function, including progenitor cell differentiation, neuronal migration and morphogenesis, and synaptic plasticity.

Human *TCF4* is expressed in the prosencephalon and the ventricular zone of the central nervous system during fetal development, and its expression remains sustained in the adult forebrain (de Pontual et al., 2009). Similarly, mouse *Tcf4* is prominently expressed in the isocortex and hippocampus during development and in adulthood (Chen et al., 2016; Jung et al., 2018). While these studies highlight broad regions in which TCF4 is particularly active, much less is known regarding the specific identity of cell types in which TCF4 is expressed. TCF4 expression has been reported in a subset of cortical neurons (Jung et al., 2018). However, it is not yet characterized which cortical neurons express TCF4, and whether brain regions outside the cortex contain TCF4-expressing cells. Moreover, TCF4-expressing hippocampal cell groups are largely unknown despite the prominent expression in the hippocampus.

Eventual pharmacological or genetic approaches to treat PTHS and other TCF4-linked disorders require knowledge of TCF4 distribution at the resolution of discrete brain areas and specific cell lineages and types. This is particularly true for gene therapy strategies that are attempting to address *TCF4* haploinsufficiency in PTHS by normalizing levels of gene expression. In order to facilitate these therapeutic efforts and further contextualize roles for TCF4 in brain development, we developed and validated a novel mouse model incorporating a Cre-dependent TCF4 green fluorescent protein (GFP) reporter. Using this line, we tracked TCF4-expressing brain regions and cell groups throughout postnatal development, with greater reliability and resolution than could previously be achieved using available antibodies (Jung et al., 2018).

## Materials and Methods

### Animals

**FIGURE 1:**
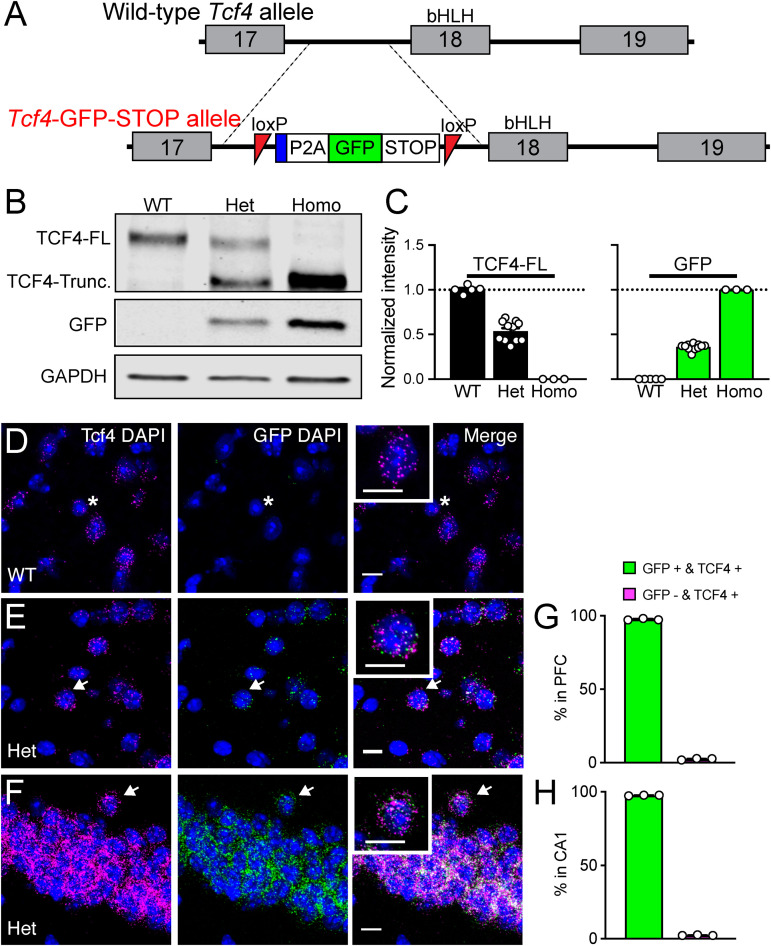
Validation of *Tcf4-LGSL* mice that faithfully report TCF4 expression. **(A)** Schematic of the strategy to generate C57BL/6J mice carrying the LoxP-P2A-GFP-STOP-LoxP cassette upstream of the basic helix-loop- helix region. Adenovirus splicing acceptor is shown by the blue box. **(B)** Representative Western blot for TCF4, GFP, and GAPDH loading control protein in embryonic brain lysates from *Tcf4^+/+^* (WT), *Tcf4*^*LGSL*/+^ (Het), and *Tcf4*^*LGSL/LGSL*^ (Homo) mice. The TCF4 antibody (recognizes mouse TCF4 aa 50–150) is designed to detect a long isoform of TCF4. We detected a TCF4 full length protein (TCF4-FL) band at approximately 76 kDa that corresponds to the long isoform in WT lysates. A TCF4 truncated protein (TCF4-Trunc.) was detected at approximately 65 kDa in Het lysates. A band for GFP or GAPDH protein was detected at approximately 26 or 35 kDa, respectively. **(C)** Quantification of Western blotting for TCF4-FL and for GFP. **(D–F)** Dual fluorescence ISH for *Tcf4* (magenta) and GFP (green) from PFC of P80 WT and *Tcf4*^*LGSL*/+^, and CA1 of *Tcf4*^*LGSL*/+^ mice. Asterisk indicates a cell expressing only *Tcf4*, and arrows indicate cells co-expressing *Tcf4* and GFP. Insets are higher magnifications. Scale bars = 10 μm. **(G,H)** Quantification of GFP-positive and -negative cells in *Tcf4*-expressing cells in the PFC and CA1 region (*n* = 3 mice). Data represent mean ± SEM.

We generated *Tcf4*^*LGSL*/+^ mice through the University of North Carolina, Chapel Hill (UNC) Animal Models Core facility. We utilized CRISPR/Cas9-mediated homologous recombination to generate *Tcf4-LoxP-GFP-Stop-LoxP* (*Tcf4*^*LGSL*^) knock-in mice on the C57BL/6J background. The *Tcf4*^*LGSL*^ allele was generated by inserting a cassette, comprised of a LoxP site, adenovirus splice acceptor, porcine teschovirus-1 2A (P2A) site, EGFP coding sequence, 3 copies of SV40 polyadenylation sequence (Stop), FRT site, and another LoxP site (Figure 1A). This cassette was inserted into *Tcf4* intron 17. The sequence of the guide RNA (gRNA) was 5′- GTCGTGCCTTACGTAGCTGGG-3.′ Mouse embryos were injected with a mixture of 400 nM Cas9 protein, 50 ng/μl *in vitro* transcribed gRNA, and 20 ng/μl supercoiled donor plasmid. The donor plasmid was constructed with 1017 bp 5′ homology arm, the LoxP-GFP-Stop-LoxP cassette, and 884 bp 3′ homology arm. Potential founder animals were screened for the presence of the insertion event by 5′ and 3′ polymerase chain reaction (PCR) assays consisting of one primer outside the targeting vector homology arms and one primer unique to the insertion event. The 5′ assay primers were Tcf4-5ScF1 (5′-GCACTTCAGGGATCGCTTA-3′) and AdSA-R2 (5′-GGGACAGGATAAGTATGACATCATC-3′), which produced a 1224 bp band. The 3′ assay primers were SV40pA-F2 (5′-GCTGATCCGGAACCCTTAAGC-3′) and Tcf4-3ScR1 (5′-CCGCCCTAATTGTTCAAAGAG-3′), which produced a 1109 bp band. Two chosen founders were checked for off-target mutations at 10 predicted off-target sites. No mutations were detected at the off-target sites screened in two founder animals. The *Tcf4*^*LGSL*/+^ knock-in mice were genotyped via PCR. The primer set of Tcf4-5ScF1 and Tcf4-3ScR1 or SV40pA-F2 and Tcf4-3ScR1 was, respectively, used to amplify the wildtype or LGSL knock-in allele.

The female *Tcf4*^*LGSL*/+^ mice were mated with heterozygous males from one of three Cre-expressing lines: *Nex-Cre* (Goebbels et al., 2006), which Klaus-Armin Nave generously provided, *Actin-Cre* (RRID:IMSR_JAX:019099), and *Gad2-Cre* (RRID:IMSR_JAX:010802). All mice were maintained on a congenic C57BL/6J background. All research procedures using mice were approved by the Institutional Animal Care and Use Committee at the UNC and conformed to National Institutes of Health guidelines.

### Western Blotting

Embryonic day 16.5–18.5 brains were dissected in ice-cold phosphate-buffered saline (PBS, pH = 7.3) and then immediately frozen with dry ice. Frozen brain samples were homogenized in glass homogenizers with ice-cold RIPA buffer [50 mM Tris–HCl, pH 7.4, 150 mM NaCl, 1% Triton X-100, 0.1% sodium dodecyl sulfate (SDS), and 0.5% Na deoxycholate] supplemented with 2 mM EDTA pH 8.0 and a protease inhibitor cocktail (Sigma, Saint Louis, MO). Tissue homogenates were cleared by centrifugation at 4°C for 20 min. Protein samples were mixed with 4x protein loading buffer (Li-COR, Lincoln, NE) and 2-mercaptoethanol (Sigma) and incubated in 95°C for 5–7 min. They were resolved by SDS-polyacrylamide gel electrophoresis and transferred to nitrocellulose membranes. Membranes were blocked for 1 h at room temperature in Odyssey blocking buffer (Li-COR) prior to incubation overnight at 4°C with primary antibodies diluted 1:500 with blocking buffer. Membranes were subsequently washed repeatedly with PBS (0.1 M Phosphate, 1.5 M NaCl) containing 0.1% Tween-20 (PBSTween) prior to incubation for 1 h at room temperature with secondary antibodies prepared in the dilution of 1:5000 in blocking buffer. The following secondary antibodies were used: donkey anti-mouse 800CW (Li-COR, 926-32212) or donkey anti-rabbit Alexa 680 (Invitrogen, A10043). Finally, blots were washed repeatedly in PBSTween followed by PBS alone prior to imaging with the Odyssey imaging system (Li-COR).

### Tissue Preparation

Postnatal mice were anesthetized with sodium pentobarbital (60 mg/kg i.p.) before transcranial perfusion with 25 ml of PBS immediately followed by phosphate-buffered 4% paraformaldehyde (pH 7.4). Brains were postfixed overnight at 4°C before 24-h incubations in PBS with 30% sucrose. Brains were sectioned coronally or sagittally at 40 μm using a freezing sliding microtome (Thermo Scientific, Kalamazoo, MI). Sections were stored at −20°C in a cryopreservative solution (45% PBS, 30% ethylene glycol, and 25% glycerol by volume).

### Histology and Immunostaining

For chromogenic staining, sections were rinsed several times with PBS, and endogenous peroxidases were quenched by incubating for 5 min in 1.0% H_2_O_2_ in MeOH, followed by PBS rinsing. Sections were washed with PBS containing 0.2% Triton X-100 (PBST) several times. Then sections were blocked with 5% normal goat serum in PBST (NGST) for 1 h at room temperature. Blocked sections were incubated in primary antibodies diluted in NGST for 24 h at 4°C. After incubation in primary antibodies, sections were rinsed several times in PBST and incubated for 1 h at room temperature in biotinylated goat anti-rabbit secondary antibodies (Vector BA-1000, Burlingame, CA) diluted 1:500 in NGST. Sections were then rinsed in PBST prior to tertiary amplification for 1 h with the ABC elite avidin-biotin-peroxidase system (Vector PK-7100). Further rinsing with PBST preceded a 3-min incubation at room temperature in 3,3′-diaminobenzidine (DAB) chromogenic substrate (0.02% DAB and 0.01% H_2_O_2_ in PBST) to visualize immune complexes amplified by avidin-biotin-peroxidase.

For immunofluorescent staining, sections were rinsed several times with PBS and PBST before blocking with NGST or 5% bovine serum albumin (BSA) in PBST for 1 h at room temperature. Sections were then incubated with primary antibodies diluted in NGST or BSA at 4°C overnight. The list of primary antibodies used is provided in Table 1. Sections were rinsed several times with PBST and then incubated with secondary antibodies for 1 h at room temperature. The following secondary antibodies from Invitrogen (Carlsbad, CA) were used at a 1:1000 dilution: goat anti-mouse Alexa 568 (A11031); goat anti-mouse Alexa 647 (A21240); goat anti-rabbit Alexa 568 (A11011); goat anti-chicken Alexa 488 (A11039); or donkey anti-goat Alexa 568 (A11057). In all experiments, 4′,6-diamidino-2-phenylindole (DAPI; Invitrogen D1306) was added during the secondary antibody incubation at a concentration of 700 ng/ml for nuclear counterstaining. Brain sections compared within figures were stained within the same experiment, under identical conditions.

**TABLE 1:** Primary antibodies used.

Antigen	Manufacturer	Dilution
APC	Millipore (Billerica, MA, United States), mouse monoclonal, clone CC-1, OP80	1:500
Calbindin	Santa Cruz (Dallas, TX, United States), goat polyclonal, sc-7691	1:500
ChAT	Millipore, goat polyclonal, AB144P	1:1,000
DARPP-32	Millipore, rabbit polyclonal, AB10518	1:1,000
GAPDH	Millipore, mouse monoclonal, clone 6C5, MAB374	1:5,000
GFAP	Dako (Glostrup, Denmark), rabbit polyclonal, Z0334	1:1,000
GFP	Novus (Centennial, CO), rabbit polyclonal, NB600-308	1:1,000
GFP	Aves Labs (Tigard, OR), chicken polyclonal, GFP-1020	1:10,000
IBA1	Wako (Osaka, Japan), rabbit polyclonal, 019-19741	1:500
NeuN	Millipore, mouse monoclonal, clone A60, MAB377	1:1,000
Olig2	Millipore, rabbit polyclonal, AB9610	1:1,000
PV	Swant (Marly, Switzerland), mouse monoclonal, PV235	1:1,000
SOM	Peninsula Laboratories (San Carlos, CA), rabbit polyclonal, T-4103	1:1,000
TCF4	Abcam (Cambridge, United Kingdom), rabbit polyclonal, ab130014. Synthetic peptide corresponds to Mouse TCF4 aa 50–150.	1:500 or 1,000
VIP	Immunostar (Hudson, WI), rabbit polyclonal, 20077	1:1,000

### *In situ* Hybridization

RNAscope Fluorescent Multiplex Assay, designed to visualize multiple cellular RNA targets in fresh frozen tissues (Wang et al., 2012), was used to detect *Tcf4* (Cat No. 423691), EGFP (Cat No. 400281-C2), *vGat* (Cat No. 319191-C3), and *vGlut1* (Cat No. 416631-C2) in mouse brain (Advanced Cell Diagnostics, Newark, CA). The target region of the *Tcf4* probe is 1120–2020 bp of mouse *Tcf4* mRNA (NM_001083967.1). Brains were extracted and frozen in dry ice. Sections were taken at a thickness of 16 μm. Staining procedure was completed to manufacturer’s specifications.

### Imaging and Figure Production

Images of brain sections stained with DAB histochemistry were obtained with a Nikon Ti2 Eclipse Color and Widefield Microscope (Nikon, Melville, NY). Images of brain sections stained by using fluorophore-conjugated secondary antibodies were obtained with Zeiss LSM 710 Confocal Microscope, equipped with ZEN imaging software (Zeiss, Jena, Germany). Images compared within the same figures were taken within the same imaging session using identical imaging parameters. Images within figure panels went through identical modification for brightness and contrast by using Fiji Image J software. Figures were prepared using Adobe Illustrator software (Adobe Systems, San Jose, CA, United States).

### Data Analysis

Images for *in situ* hybridization (ISH) colocalization analysis were captured from consistent coronal section planes across different mouse brains (PFC, STR: ∼ 1.10 mm; CA1, BLA, TH: ∼−2.06 mm; VC: ∼−2.70 mm from bregma). The DAPI image from each brain region (265.69 × 265.69 μm) was converted to 8-bit in black and white, and its threshold was adjusted using the Huang method built into Fiji software. For the image with *Tcf4* or GFP staining, the ISODATA threshold method was consistently applied. To identify mean *Tcf4* or GFP fluorescence intensity level for each nucleus (DAPI), we used CellProfiler software, which is a free open-source software that allows one to measure and analyze cell images automatically (Kamentsky et al., 2011).

## Results

### Validation of *Tcf4-LGSL* Mouse Model

To investigate the spatiotemporal profile of TCF4-expressing cells, we engineered mice with a LoxP-GFP-STOP-LoxP (LGSL) cassette introduced into intron 17 of the *Tcf4* allele (Figure 1A). An adenovirus splicing acceptor was included in the cassette to avoid alternative splicing of intron 17 (Figure 1A). This design allowed us to examine TCF4-expressing cells with high confidence, as GFP can be detected by commercial antibodies. Moreover, the insertion of a 2A self-cleaving peptide (P2A) enables GFP molecules to freely diffuse throughout the cytoplasm, making it possible to track axonal projections from TCF4-expressing neurons, though at the cost of not being able to use it to identify the subcellular localization of TCF4. The GFP and STOP cassette is flanked by LoxP sites, enabling their Cre-mediated deletion, and in turn, reinstating the capacity to express full-length, functional TCF4 from the locus.

As predicted from our design, brain lysates from *Tcf4^+/+^* (WT) mice produced a single full-length TCF4 band by Western blot, whereas lysates from *Tcf4*^*LGSL*/+^ (Het) mice produced both the full-length and truncated TCF4 protein, and lysates from *Tcf4*^*LGSL/LGSL*^ (Homo) mice produced only a truncated TCF4 band (Figure 1B). GFP was present only in *Tcf4*^*LGSL*/+^ and *Tcf4*^*LGSL/LGSL*^ lysates (Figure 1B). The band intensity of full-length TCF4 was reduced by approximately half in lysates from *Tcf4*^*LGSL*/+^ compared to WT mice (Figure 1C: WT: 1.00 ± 0.02, *n* = 5; Het: 0.54 ± 0.03, *n* = 11; Homo: 0.00 ± 0.00, *n* = 3). GFP levels were higher in lysates from *Tcf4*^*LGSL/LGSL*^ compared to *Tcf4*^*LGSL*/+^ mice (Figure 1C: WT: 0.00 ± 0.00; *n* = 5, Het: 0.36 ± 0.01, *n* = 11; Homo: 1.00 ± 0.00, *n* = 3). These results validated that the LGSL cassette produced GFP and truncated TCF4 protein.

To verify that GFP faithfully reports TCF4 expression, we performed dual ISH using probes specific to *Tcf4* or GFP mRNA. GFP signals were detected in cells from adult *Tcf4*^*LGSL*/+^ mice, but absent in cells from WT mice (Figure 1D), proving the specificity of the GFP probe detection. *Tcf4* signals were observed in both WT and *Tcf4*^*LGSL*/+^ mice (Figures 1E,F). Quantification of cells expressing both GFP and *Tcf4* revealed an approximate 97% overlap (Figures 1G,H: prefrontal cortex (PFC): 97.56 ± 0.31 %; CA1: 97.58 ± 0.13 %), as only 2.4 % of *Tcf4-*expressing cells lacked detectable GFP mRNA (Figures 1G,H: PFC: 2.44 ± 0.32 %; CA1, 2.42 ± 0.13 %). These results verify that the GFP expression in *Tcf4*^*LGSL*/+^ mice faithfully reports *Tcf4* expression.

### Comparison of GFP Reporter and TCF4 Antibodies

**FIGURE 2:**
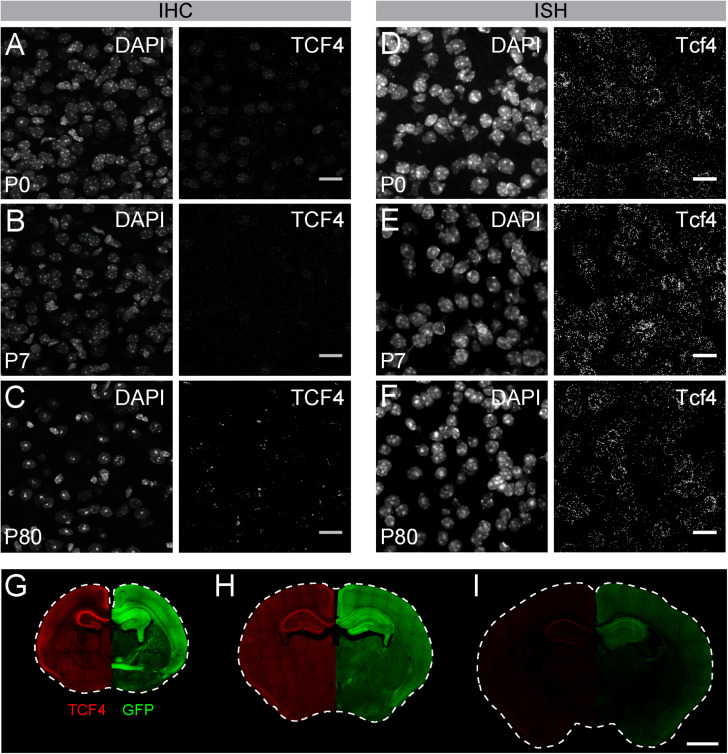
GFP reporter enhances sensitivity to detect TCF4 by immunohistochemistry. **(A–C)** Immunostaining and **(D–F)** ISH of TCF4/*Tcf4* and DAPI from P7, P15, and P80 mouse cortex. Immunostaining shows decreased detection of TCF4 protein using TCF4 antibody, whereas ISH shows comparable number of *Tcf4* expressing cells during postnatal development. Scale bars = 20 μm. **(G–I)** Dual immunostaining of P0, P7, and P80 using TCF4 and GFP antibodies in coronal sections from *Tcf4*^*LGSL*/+^ mice. Image is taken from the same double-labeled section. Scale bar = 1 mm.

Of commercially available TCF4 antibodies, only one has been validated for immunostaining using homozygous *Tcf4* knock-out tissues (Jung et al., 2018). We used this antibody to visualize TCF4-expressing cells in WT brain. We observed weak protein signals in brain cell nuclei at postnatal day (P) 7 (Figure 2A). Under identical experimental conditions, we failed to detect appreciable TCF4 protein signals at P15 and P80 (Figures 2B,C). TCF4 expression may dwindle to undetectable levels, or cease altogether, over the course of brain maturation. To distinguish between these possibilities, we performed ISH for *Tcf4* in age-matched WT brains. We observed comparable numbers of *Tcf4-*expressing cells between neonatal and adult brains (Figures 2D–F), indicating expression of *Tcf4* transcript persisted in most cells across postnatal development, albeit likely at reduced levels. Thus, failure to immunodetect TCF4 protein in adult brain is due to the limited sensitivity of the TCF4 antibody, not the absence of the target protein.

To directly compare sensitivities for detecting TCF4 and GFP antibodies, we performed double immunohistochemistry in brain sections of *Tcf4*^*LGSL*/+^ mice, from birth into adulthood. GFP and TCF4 labeling patterns were similar across postnatal development, though GFP labeling was of visually greater intensity than TCF4 labeling (Figures 2G–I). The disparity in labeling intensity was also apparent at P10 and was even more pronounced by adulthood when TCF4 labeling outside of the hippocampus was barely detectable (Figures 2H,I). We also detected GFP labeling within axonal projections (Figure 2G). These data highlight advantages of the GFP reporter — increased sensitivity and the capacity to track the axonal projections of TCF4-expressing neurons—for mapping TCF4 expression patterns across all postnatal ages.

### TCF4 Expression Patterns of the Adult Mouse Brain

**FIGURE 3:**
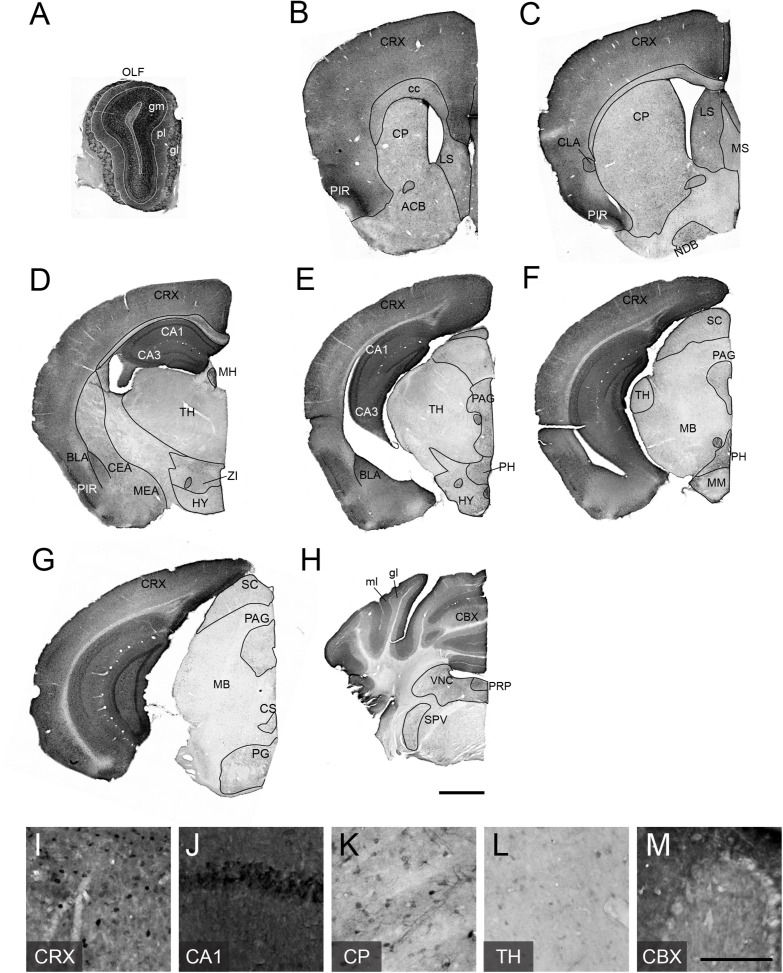
TCF4 expression patterns of adult mouse brain. **(A–H)** DAB immunostaining of GFP (for TCF4) in coronal brain sections of adult *Tcf4*^*LGSL*/+^ mice. **(I–N)** High magnification view of CRX, CA1, CP, TH, and CBX. TCF4-expressing cells are prominently found in CRX, CA1, and CBX. Scale bars = 1 mm and 200 μm for higher magnification insets. The list of abbreviations used is provided in Table 2.

To examine adult patterns of TCF4 expression, we stained for GFP across the rostral to caudal extent in coronal sections from *Tcf4*^*LGSL*/+^ mice (Figures 3A–H). We observed the most prominent GFP labeling intensity in the pallial region, which contains the olfactory bulb, cortex, and hippocampus (Figures 3A–G). Cells in the glomerular (gm), external plexiform (pl), and granule layers (gr) of the olfactory bulb (OLF) were strongly labeled with GFP (Figure 3A). Throughout the entire cortex, intense GFP staining was seen in almost all areas and in every layer (Figures 3B–G,I). Expression was strong in the hippocampus, especially in the pyramidal cell layer of Ammon’s horn (Figure 3J), and in the cerebellum, highlighted by concentrated GFP labeling in the molecular (mo) and granule cell (gl) layers (Figures 3H,M).

**TABLE 2:** Abbreviations.

ACB	Nucleus accumbens
BLA	Basolateral amygdalar nucleus
CA1	Cornu ammonis1
CA3	Cornu ammonis3
CBX	Cerebellum
CC	Corpus callosum
CEA	Central amygdalar nucleus
CLA	Claustrum
CP	Caudate putamen
CRX	Cortex
CS	Superior central nucleus raphe
egl	External granule layer of cerebellum
gl	Granule layer of cerebellum
gm	Glomerular layer of olfactory bulb
gr	Granule layer of olfactory bulb
HY	Hypothalamus
igl	Inner granule layer of cerebellum
LSr	Lateral septal nucleus, rostral (rostroventral) part
MB	Midbrain
MEA	Medial amygdalar nucleus
MH	Medial habenula
ml	Molecular layer of cerebellum
MM	Medial mammillary nucleus
MS	Medial septal nucleus
NDB	Diagonal band nucleus
OLF	Olfactory bulb
PAG	Periaqueductal gray
PFC	Prefrontal cortex
PG	Pontine gray
PIR	Piriform area
pl	Plexiform layer of olfactory bulb
PRP	Nucleus prepositus
SC	Superior colliculus
SPV	Spinal nucleus of the trigeminal
STR	Striatum
TH	Thalamus
VC	Visual cortex
VNC	Vestibular nuclei
ZI	Zona incerta

While the entire pallial region and cerebellum stained intensely for GFP, subsets of other brain regions were lightly and sparsely labeled for GFP. In the pallial derivatives, cells in the basolateral amygdala nucleus (BLA) and claustrum (CLA) were stained for GFP. In the subpallial derivatives, we detected GFP-positive cells in the central amygdala nucleus (CEA) and medial amygdala nucleus (MEA) (Figures 3C–E). We also noted GFP labeling of cells in the caudoputamen (CP), nucleus accumbens (ACB), lateral septal nucleus (LS), medial septal complex (MS), and nucleus of the diagonal band (NDB) (Figures 3B,C,K), although this labeling was much lighter, and the stained cell density was much lower than what we observed in the pallial region. In the hypothalamus, we observed the highest density of GFP-expressing cells in posterior hypothalamic nucleus (PH) (Figures 3D–E). In the diencephalic prosomeres, the medial habenula (MH) stood out for its strong GFP labeling intensity (Figure 3D), contrasting sharply with other thalamic nuclei that were generally devoid of detectable GFP (Figures 3D,E,L). In the prethalamic structure, we observed GFP-positive cells in zona incerta (ZI). In the midbrain, GFP labeled cells in periaqueductal gray (PAG) and superior colliculus (SC) (Figures 3E–G). In the hindbrain, we observed GFP-expressing cells in the superior central nucleus raphe (CS), pontine gray (PG), vestibular nuclei (VNC), nucleus prepositus (PRP), and spinal nucleus of the trigeminal (SPV) (Figures 3G,H).

**FIGURE 4:**
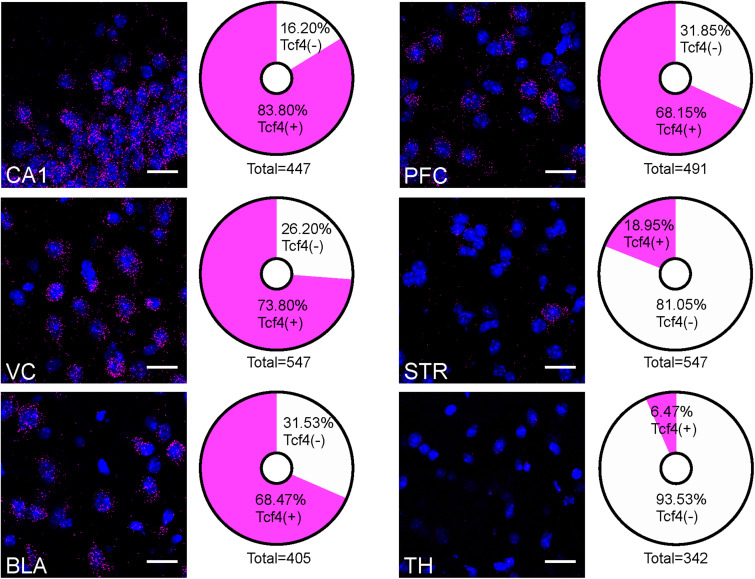
Quantification of *Tcf4*-expressing cells in multiple brain regions of adult WT brain. Representative ISH images of *Tcf4* and DAPI and proportionality of *Tcf4*-positive (magenta) and -negative (white) cell populations in CA1, VC, BLA, PFC, STR, and TH. *Tcf4* mRNA is present at high levels in the CA1, VC, BLA, and PFC. The total numbers in the pie chart center represent the quantified DAPI cells per brain region. Values represent the mean percentages. *n* = 3 mice. Scale bars = 20 μm.

The contrast in labeling intensity of GFP detected in the pallial region along with cerebellum and the rest of the brain suggests differences in TCF4-expressing cell densities. To compare the expression across different brain regions, we fluorescently labeled *Tcf4* in adult WT tissues via ISH and quantified *Tcf4*-containing cells. We detected *Tcf4* signals in all examined brain regions, including CA1, visual cortex (VC), BLA, PFC, CP, and TH (Figure 4). Consistent with our qualitative observations of GFP labeling intensity (Figure 3), the percentage of cells expressing *Tcf4* transcript was dramatically higher in CA1, VC, BLA, and PFC compared to CP and TH (Figure 4).

### TCF4 Expression Patterns of the Neonatal and Juvenile Mouse Brain

**FIGURE 5:**
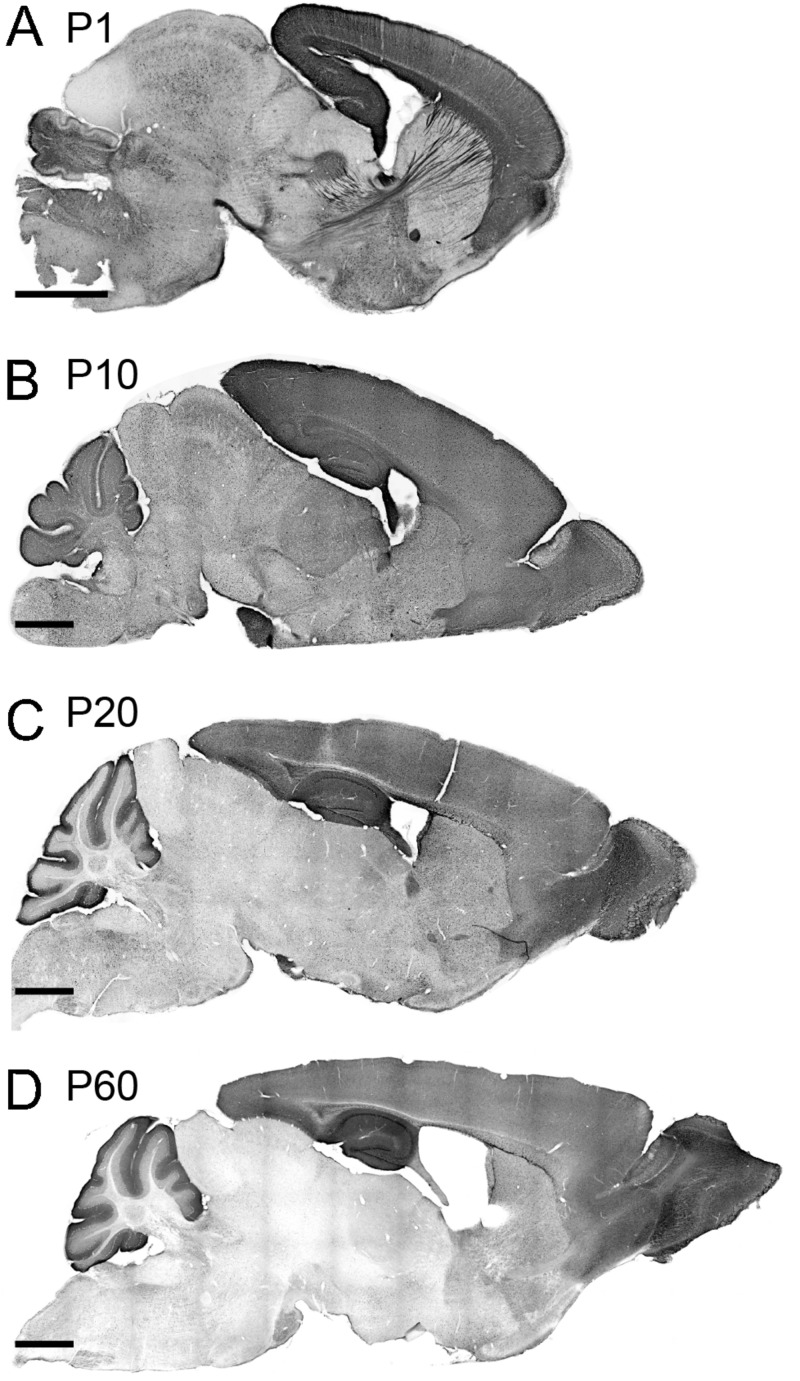
TCF4 expression patterns of the neonatal and juvenile mouse brain. **(A–D)** DAB immunostaining of GFP (for TCF4) in sagittal brain sections of *Tcf4*^*LGSL*/+^ mice at P1, P10, P20, and P60. A similar staining pattern largely persists throughout postnatal development. Scale bars = 1 mm.

We investigated the spatial dynamics of TCF4 expression during postnatal brain development by examining GFP reporter expression at P1, P10, P20, and P60. At P1, the pallial region stood out with the strongest GFP staining. Other derivatives from prosencephalon, mesencephalon, and rhombencephalon were also stained for GFP. Cell densities were lower in these derivatives than the pallial region. The lowest level of GFP expression was detected in the thalamus and inferior colliculus. Intensely labeled axonal projections were unique to the P1 timepoint. Most notably, some GFP-stained axons were extended from the cortical neurons into discrete thalamic nuclei. Other GFP-stained cortical axons were extended to invade the hypothalamus and pons (Figure 5A). We also detected the cerebral peduncle intensely stained for GFP. These labeling patterns demonstrate that, at an early postnatal stage, corticothalamic and subcerebral projection neurons expressed TCF4. Additionally, axons coursing through the corpus collosum, fimbria, internal capsule, fornix, and anterior commissure were labeled strongly for GFP (Figures 5A, 2G). GFP expression remained high in the pallial region and cerebellum at P10. We also detected GFP-expressing cells throughout the hypothalamus, midbrain, and hindbrain. Strikingly, GFP expression level was slightly increased in the thalamus at this age compared with P1 (Figure 5B). This slight increase is potentially caused by axonal fibers spreading into the midline nuclei. A similar pattern of corticothalamic fibers was reported at this age in transgenic mice that drive GFP in early cortical preplate and subplate neurons (Jacobs et al., 2007). At P20, GFP expression level was reduced in the thalamus, hypothalamus, midbrain, and hindbrain. The pallial region, cerebellum, and some hindbrain and hypothalamic nuclei were intensely stained for GFP (Figure 5C). The expression pattern observed in P20 brain was conserved in P60 brain, although the overall expression level of P60 brain was slightly decreased compared with P20 brain. Our data show that high levels of GFP labeling were persistently detected in the pallial region and cerebellum in all ages (Figure 5). These data suggest that TCF4 could be involved in early stages of neuronal development across the entire brain, but as the brain matures, TCF4 function becomes increasingly restricted to the pallial region and cerebellum.

### Glutamatergic and GABAergic Cells, Astrocytes, and Oligodendrocytes Express TCF4 in the Prefrontal Cortex

**FIGURE 6:**
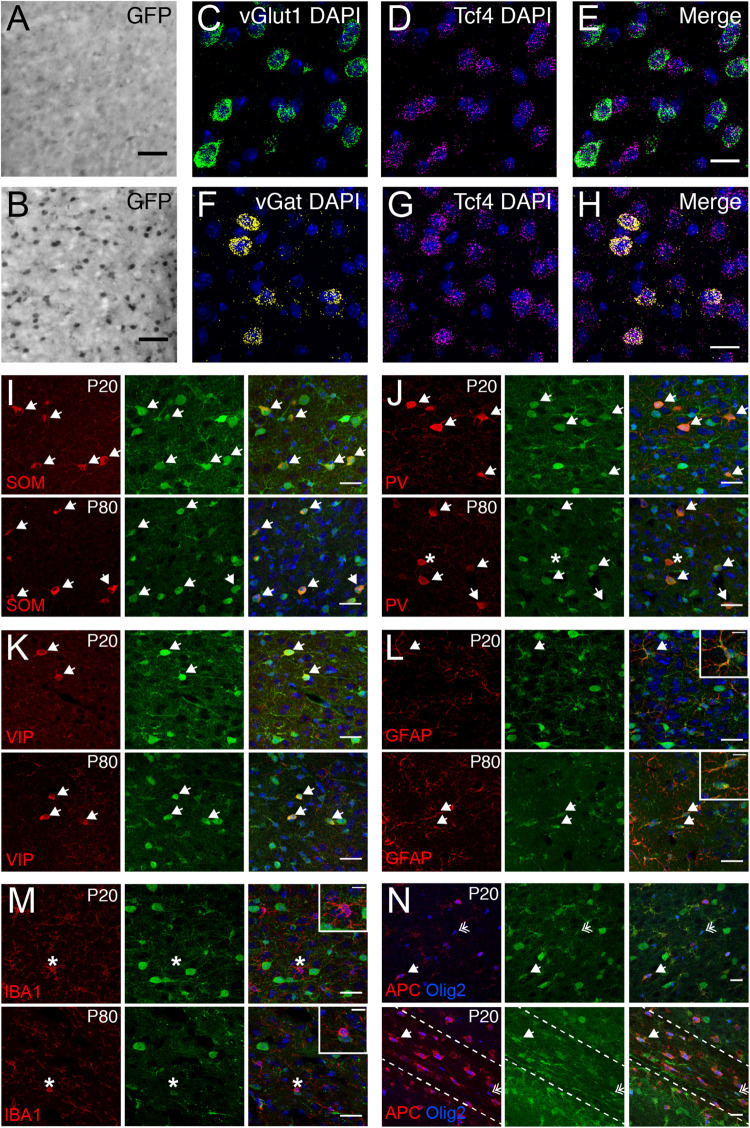
Glutamatergic, GABAergic cells, astrocytes, and oligodendrocytes express TCF4 in the PFC. **(A,B)** DAB immunostaining of GFP (for TCF4) in coronal sections of P80 *LGSL::Gad2-cre* or *LGSL::Nex-cre* mice where GFP protein is deleted in inhibitory or excitatory neurons, respectively. Both glutamatergic and GABAergic cells express TCF4. Scale bars = 0.5 mm. **(C–E, F–H)** Dual ISH for *vGult1* and *Tcf4* and for *vGat* and *Tcf4* in P80 WT brain tissue, confirming that *Tcf4* mRNA is present in *vGlut1*- and *vGat*-expressing cells. Scale bars = 20 μm. **(I–K)** Dual immunostaining of interneuron subtype-specific markers, SOM, PV, or VIP, and GFP (for TCF4) in P20 and P80 *LGSL::Nex-cre* mice. TCF4 is expressed in nearly all SOM-, PV-, and VIP-positive interneurons (arrows). Asterisk represents rare interneuron that does not express GFP. Scale bars = 30 μm. **(L,M)**: Dual immunostaining of astrocyte marker, GFAP, or microglial marker, IBA1, and GFP (for TCF4) in P20 and P80 *LGSL::Nex-cre* mice. GFAP-labeled cells express GFP (arrows), but IBA1-labeled cells do not express GFP (asterisk). Scale bars = 30 or 10 μm for higher magnification insets. **(N)** Triple immunostaining of APC, Olig2, and GFP (for TCF4) in the PFC (top panel) and corpus callosum (bottom panel) of P20 *LGSL::Nex-cre* mice. TCF4 is expressed in mature (arrow) and immature (double arrow) oligodendrocytes. Scale bars = 20 μm.

We used the GFP reporter line to characterize the cell type-specific expression of TCF4 in the PFC. GABAergic and glutamatergic neurons represent two major neuronal classes that we could more easily distinguish upon reciprocal Cre deletion, which succeeded in eliminating expression of the GFP reporter one class at a time. We generated *LGSL::Gad2-Cre* mice to delete GFP expression from GABAergic neurons (Taniguchi et al., 2011). We detected relatively light GFP staining in putative glutamatergic neurons throughout the cortical layers (Figure 6A). We also generated *LGSL::Nex-Cre* mice to delete GFP selectively from forebrain glutamatergic neurons (Goebbels et al., 2006). We detected strong residual labeling in GABAergic cells (Figure 6B). To confirm that *Tcf4* expression is ubiquitous in these neuronal classes, we performed double ISH in adult WT PFC for *Tcf4* in combination with either *vGlut1* or *vGat*, which encode the vesicular transporters for glutamate and GABA, respectively. We found almost all *vGlut1*- and *vGat*-expressing cells contained *Tcf4* (Figures 6C–H). These findings suggested that TCF4 may be ubiquitously expressed in cortical glutamatergic and GABAergic cell populations.

Nearly all cortical GABAergic interneurons belong to one of three groups defined by the expression of parvalbumin (PV), somatostatin (SOM), and the ionotropic serotonin receptor 5HT3a (5HT3aR) (Rudy et al., 2011). Each group differs in its morphological and electrophysiological properties and plays unique roles in cortical circuit function (DeFelipe, 1993; Gonchar and Burkhalter, 1997; Markram et al., 2004). To determine whether TCF4 is expressed in specific GABAergic interneuron subtypes, we performed coimmunostaining for GFP and representative subgroup-specific markers in the juvenile and adult *LGSL::Nex-Cre* mice. There are currently no suitable antibodies for staining 5HT3aR, so we chose vasoactive intestinal peptide (VIP) as an alternative marker, which is expressed by approximately half of all 5HT3aR-expressing neurons (Lee et al., 2010; Rudy et al., 2011). We found that nearly all SOM, PV, and VIP labeled interneurons were copositive with GFP in the PFC (Figures 6I–K) at P20 and P80, suggesting that TCF4-expressing GABAergic cells consist of SOM, PV, and VIP interneurons.

Over the course of our study, we observed that a subset of GFP-stained cells did not stain positive for NeuN (data not shown), indicating that TCF4 may be expressed in glial cell populations. We costained for GFP and either the astrocyte marker glial fibrillary acid protein (GFAP), or the microglia marker ionized calcium binding adaptor molecule 1 (IBA1), in *LGSL::Nex-Cre* mice. GFP/GFAP copositive astrocytes were present throughout the PFC of both juvenile and adult mice (Figure 6L). However, GFP-stained glia did not costain for IBA1 (Figure 6M). Due to the recently established role for TCF4 in regulating the maturation of oligodendrocyte progenitors (Phan et al., 2020), we expected that TCF4 would be expressed in oligodendrocyte lineage cells. Olig2 marks all stages of oligodendrocyte lineage, and APC (or CC1) marks the maturational process (Bhat et al., 1996). The majority of Olig2/APC positive cells, reflecting mature, myelinating oligodendrocytes, stained for GFP in the PFC and corpus callosum at P20 (Figure 6N). Similarly, a subset of immature oligodendrocytes, labeled only by Olig2, stained for GFP (Figure 6N). Our results show that among major glial cell populations in the brain, astrocytes and both immature and mature oligodendrocytes express TCF4, while microglia appear to lack TCF4 expression.

### Pyramidal Cells, GABAergic Interneurons, and Astrocytes Express TCF4 in the Hippocampus

**FIGURE 7:**
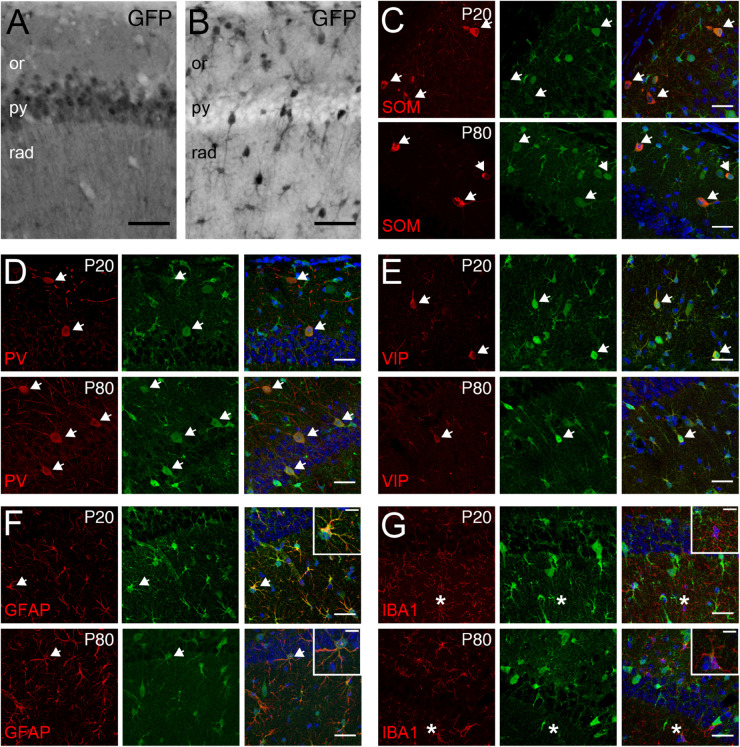
Pyramidal cells, GABAergic interneurons, and astrocytes express TCF4 in the hippocampus. **(A,B)** DAB immunostaining of GFP (for TCF4) in the CA1 of P80 *LGSL::Gad2-cre* or *LGSL::Nex-cre* mice. Both pyramidal layer cells and GABAergic cells express TCF4. Scale bars = 0.5 mm. **(C–E)** Dual immunostaining of interneuron subtype-specific markers, SOM, PV, or VIP, and GFP (for TCF4) in P20 and P80 *LGSL::Nex-cre* mice. TCF4 is expressed nearly all SOM-, PV-, or VIP-positive interneurons (arrows). **(F,G)** Dual immunostaining of GFAP or IBA1, and GFP (for TCF4) in P20 and P80 *LGSL::Nex-cre* mice. GFAP-labeled cells express GFP (arrow), but IBA1-labeled cells do not express GFP (asterisks). Scale bars = 30 or 10 μm for higher magnification insets.

*Tcf4* deficient mice exhibited deficits in the behavioral tasks that require proper hippocampal functions. Additionally, a form of hippocampal synaptic plasticity was altered in these mice (Kennedy et al., 2016; Thaxton et al., 2018). Therefore, we characterized TCF4-expressing cell types in this brain region to reveal which cell types might contribute to these phenotypes. First, we examined glutamatergic and GABAergic cell populations by staining for GFP in *LGSL::Gad2-Cre* and *LGSL::Nex-Cre* mice. As expected from our ISH data (CA1, Figure 4), glutamatergic pyramidal cells of the CA1 region exhibited strong GFP labeling (Figure 7A). Moreover, we detected strong residual labeling in GABAergic cells across the layers (Figure 7B). The hippocampal GABAergic inhibitory circuits consist of SOM-, PV-, VIP-, neuropeptide Y-, calretinin-, and cholecystokinin-expressing interneurons (Pelkey et al., 2017). We tested whether some of these inhibitory interneurons expressed TCF4 by performing coimmunostaining in *LGSL::Nex-Cre* brain. We found that SOM-, PV- and VIP-positive neurons stained for GFP at P20 and P80 (Figures 7C–E). GFP staining in *LGSL::Nex-Cre* mice revealed clearly identifiable star-shaped cells (rad. layer, Figure 7B). Our coimmunostaining result showed that GFAP-positive astrocytes stained for GFP (Figure 7F). But, IBA-positive microglial cells were devoid of GFP (Figure 7G). Our results demonstrated that TCF4-expressing hippocampal cell groups consist of astrocytes, pyramidal cells, and SOM-, PV-, and VIP-containing interneurons.

### SOM and PV Interneurons and Astrocytes Express TCF4 in the Striatum

**FIGURE 8:**
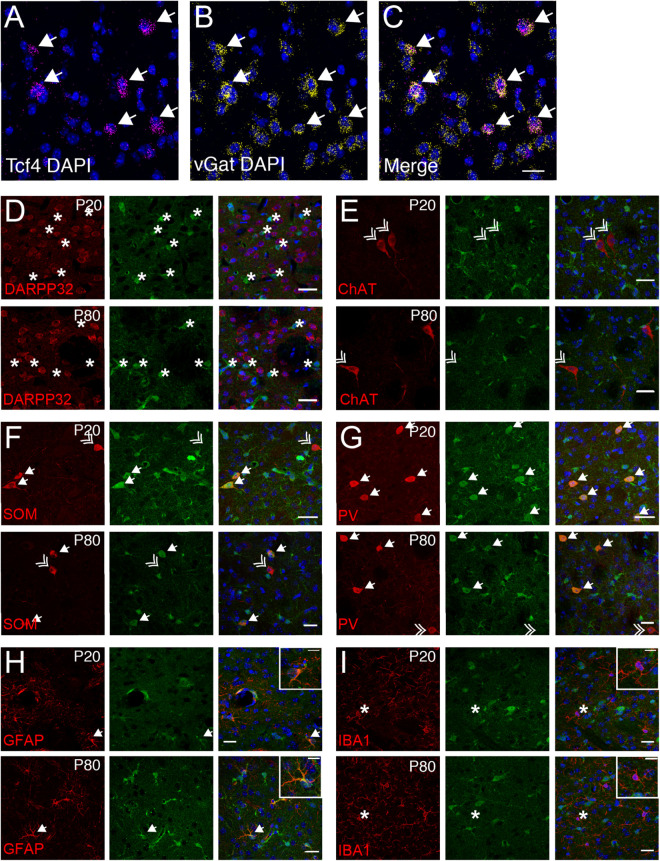
Striatal interneurons, but not medium spiny neurons, express TCF4. **(A–C)** Representative ISH images for *Tcf4* and *vGat* from adult WT striatum, showing that specific subtypes of interneurons express *Tcf4* (arrows). Scale bar = 20 μm. **(D–G)** Dual immunostaining of DARPP32, ChAT, SOM, or PV and GFP (for TCF4) in P20 and P80 *Tcf4*^*LGSL*/+^ mice. The representative staining images reveal that SOM- and PV-positive subtype interneurons express TCF4 (arrow). Asterisks represent only GFP-positive neurons. Double arrows represent interneuron subtypes that do not express GFP. Scale bars = 20 μm. **(H,I)** Dual immunostaining of GFAP or IBA1, and GFP (for TCF4) in P20 and P80 Tcf4^*LGSL*/+^ mice. GFAP-labeled cells express GFP (arrow), but IBA1-labeled cells do not express GFP (asterisk). Scale bars = 30 or 10 μm for higher magnification insets.

The vast majority of striatal neurons signal through GABA to inhibit their target cells (Koos and Tepper, 1999; Gittis et al., 2010). Because we observed that only ∼19% of striatal cells express *Tcf4* (STR, Figure 4), we speculated that these would comprise specific subgroups of GABAergic neurons. Using double ISH, we detected *Tcf4* signals in a subset of *vGat*-expressing cells (Figures 8A–C). We subsequently employed a double immunostaining approach in juvenile and adult *Tcf4*^*LGSL*/+^ mice to further define TCF4-expressing GABAergic population. We found that the GFP-labeled cells were not colocalized with medium spiny neurons (MSNs), marked by DARPP32 (Figure 8D), indicating that GABAergic MSNs do not express TCF4. Cholinergic interneurons, marked by choline acetyltransferase (ChAT), represent another major cell GABAergic class in the striatum in which GFP was not expressed (Figure 8E). SOM and PV expression characterizes other GABAergic interneuron types in the striatum (Munoz-Manchado et al., 2018). We detected GFP in SOM- and PV-positive interneurons at P20, and this colocalization persisted in the adult striatum (Figures 8F,G). Interestingly, a few SOM or PV positive cells did not stain for GFP, raising the possibility that TCF4 expression could confer unique functional properties to subsets of PV and SOM interneurons. We showed earlier in this study that TCF4 was expressed in astrocytes, but not microglial cells, in the cortex and hippocampus (Figures 6L,M, 7F,G). Thus, we asked whether this expression pattern also applied to the striatum. We detected GFP in GFAP-positive cells, but not in IBA1-positive cells (Figures 8F,G). Collectively, these data suggest that TCF4 expression in the striatum is restricted to PV and SOM interneurons and astrocytes, but not to medium spiny, cholinergic neurons, and microglial cells.

### TCF4 Is Enriched in the Molecular and Granule Cell Layer of the Cerebellar Cortex

**FIGURE 9:**
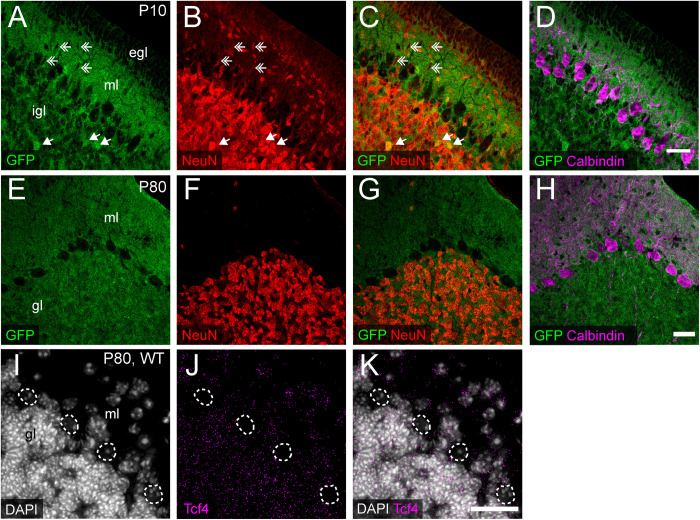
Cerebellar granule and molecular layer cells, but not Purkinje cells, express TCF4. **(A–H)** Triple immunostaining of GFP (for TCF4), NeuN, and Purkinje cell marker, calbindin, in P20 and P80 *Tcf4*^*LGSL*/+^ mouse cerebellum. The representative images confirm that migrating NeuN-positive granule cells in the molecular layer (ml) lack TCF4 (double arrows), and post-migratory granule cells in the inner granule layer (igl) express TCF4 (arrows). Purkinje cells do not express TCF4. egl = External granule layer. **(I–K)** Representative ISH images for *Tcf4* and DAPI in WT adult cerebellum, showing that *Tcf4* mRNA is present in granule and molecular layer (gl and ml) cell nuclei, but it is absent in Purkinje cell nuclei (dashed line). Scale bars = 30 μm.

We consistently observed strong GFP immunoreactivity in the cerebellum across postnatal development (Figures 3M, 5). Thus, we further characterized TCF4 distribution in this structure, focusing on the molecular, Purkinje cell, and granule cell layers. At P10, a timepoint of ongoing cerebellar histogenesis (Altman, 1969), we found that GFP was enriched in the extracellular area of the molecular layer and inner granule layer, but absent in the external granule layer and Purkinje cell layer (Figure 9A). NeuN staining clearly marked neurons with a multipolar morphology, presumably traversing the molecular layer toward the inner granule layer (Figure 9B). These cells were negative for GFP (Figures 9A–C), suggesting that migrating granule cells do not express TCF4. In the inner granule layer, where post-migratory granule cells undergo maturation, we infrequently found NeuN-positive cells that costained with GFP (Figures 9A–C). By adulthood, however, nearly all NeuN-positive neurons in the granule layer costained for GFP (Figures 9E–G), leading us to surmise that cerebellar granule cells only upregulate TCF4 expression as they mature. Regardless of age, GABAergic Purkinje cell bodies, labeled by calbindin, lacked GFP staining (Figures 9D,H). Consistent with our GFP immunostaining results, ISH for *Tcf4* in adult wildtype cerebellum confirmed that most granule cells expressed *Tcf4*, while GABAergic Purkinje cells did not (Figures 9I–K). We also detected *Tcf4*-expressing cells in most GABAergic interneurons of the molecular layer (Figures 9I–K).

## Discussion

It is imperative to understand the cellular distribution of TCF4 during postnatal development in order to guide the delivery of therapeutics for TCF4-linked disorders. Toward this goal, we developed a mouse with a TCF4-GFP reporter that conferred greater sensitivity for detecting TCF4 expression than existing antibody detection methods (Figure 2). We validated the TCF4-GFP reporter mouse model by using double *in situ* labeling to show that about 98% of *Tcf4*-containing cells express GFP, proving the mouse model as a faithful reporter for TCF4 (Figures 1E–H). While the GFP reporter was designed to diffuse freely through the cytoplasm, and thus is not a marker of TCF4 subcellular localization, the reporter offers the advantage that it can label dendritic arborizations and axonal projections of TCF4-expressing neurons (Figures 2G, 5A). To improve our ability to observe TCF4-expressing cell types, we conditionally deleted the GFP reporter in a Cre-dependent manner. This allowed us to more easily observe the remaining GFP-positive cells with an improved signal to noise ratio (Figures 6A,B, 7A,B). We used these approaches, coupled with double-labeling immunohistochemistry and *in situ* hybridization, to characterize the cell type-specific and spatiotemporal expression of TCF4 in the postnatal mouse brain.

### TCF4 Expression Patterns and Their Implications in Pathology of TCF4-Linked Disorders

Common genetic variants in and around *TCF4* are associated with a range of neurodevelopmental and psychiatric disorders. Rare *TCF4* single nucleotide variants have been described in schizophrenia patients whose symptoms include impairments of attention, memory, social cognition, and executive functions (Basmanav et al., 2015; Forrest et al., 2018). *TCF4* mutations have been found in large-scale genotyping studies in patients with intellectual disability and autism spectrum disorder (ASD) (Kharbanda et al., 2016; Maduro et al., 2016). Haploinsufficiency of *TCF4* causes PTHS – a rare form of intellectual disability associated with characteristic facial features and motor and speech dysfunction (Goodspeed et al., 2018; Zollino et al., 2019). Collectively, these studies implicate TCF4 in a range of brain disorders that are commonly associated with cognitive dysfunction. The prefrontal cortex is linked with a range of cognition including cognitive control, lower-level sensory processing, memory, and motor operations (Miller, 2000). The hippocampus supports learning and memory functions in a spatiotemporal context (Dupret et al., 2010; Rubin et al., 2014). The prefrontal cortex and hippocampus are thus suspected pathophysiological loci for TCF4-linked disorders. TCF4 is enriched in most cortical and hippocampal cells, including excitatory and inhibitory neurons, as well as astrocytes, and oligodendrocytes, in the juvenile and adult mouse brain (Figures 5–7). These findings in TCF4-expressing cell groups support the idea that functions of the prefrontal cortex and hippocampus are particularly susceptible to subtle changes in TCF4 expression. TCF4 loss is associated with defects in cortical cell positioning, dendritic spines, and arborizations (Chen et al., 2016; Li et al., 2019). TCF4 haploinsufficiency results in reduced hippocampal volume and cortical thickness in mice (Jung et al., 2018). These structural phenotypes are likely linked to functional consequences, including abnormal neuronal excitability and synaptic plasticity in the prefrontal cortex and hippocampus, which are consistently observed across multiple PTHS mouse models (Kennedy et al., 2016; Rannals et al., 2016; Thaxton et al., 2018). These cell physiological defects in turn likely contribute to the impairments in cognition and memory functions in patients with TCF4-linked disorders.

Severe motor delay and stereotypic behavior are consistent phenotypes observed in patients with PTHS (Goodspeed et al., 2018; Zollino et al., 2019). However, the potential mechanism underlying motor deficits and stereotypies remains unknown. The striatum is involved in translating cortical activity into adaptive motor actions and controlled movement (Kreitzer and Malenka, 2008). At the circuit levels, some striatal interneurons receive direct cortical afferents. For example, activity of striatal PV interneurons, known to inhibit MSNs, are enhanced by cortical stimulation. Regardless of cortical projections, SOM interneurons locally target MSNs and ChAT-positive neurons (Straub et al., 2016). TCF4 is expressed in PV and SOM interneurons, but not in MSNs and ChAT-positive neurons (Figure 8), suggesting that TCF4 loss may alter striatal circuit functions through PV and SOM interneurons. Disruptions in GABAergic circuits of the striatum have been found in neuropsychiatric disorders and autism (Maia and Frank, 2011; Rapanelli et al., 2017; Skene et al., 2018). Further experiments will be required to determine whether GABAergic circuit dysfunction occurs with TCF4 loss, and if so, whether it is the direct cause of motor delay and stereotypic behaviors.

The cerebellum contributes to motor coordination, cognitive processing and emotional control (Schmahmann and Caplan, 2006). It is structurally and functionally abnormal in patients diagnosed with ASD and other neurodevelopmental disorders (Rogers et al., 2013). Cognitive functions are impaired in individuals with developmental reductions in cerebellar volume. Also, the degree of volume reduction is correlated with the degree of cognitive impairment (Steinlin, 2008; Bolduc et al., 2012). Patients with PTHS display reduced volume of the cerebellum (Peippo et al., 2006; Whalen et al., 2012), which may contribute to severity of cognitive and motor impairment. The adult human cerebellum expresses high levels of TCF4 (Jung et al., 2018). Similar to the human brain, TCF4 is prominently expressed in the mouse cerebellum during postnatal development and in adulthood (Figures 3H, 5). Our data thus suggest that the cerebellum is a candidate brain region that needs to be evaluated to determine whether TCF4 regulates cerebellar structure, and perhaps function. We found that differentiated and migrating granule cells repress TCF4 expression, while post-migratory mature granule cells upregulate TCF4 expression (Figures 9A–C,E–G). Our findings indicate that TCF4 is positioned to modulate maturation of the granule cells after migration. Future study will need to address whether TCF4 loss or dysfunction alters cerebellar anatomy and local circuit function, and if so, whether changes in cerebellar circuit directly cause motor and cognitive deficits.

Neurons are produced in the proliferative ventricular zone (VZ) and the subventricular zone (SVZ) of the embryonic telencephalon during development of the cortex (Bystron et al., 2008). These neurons migrate along radial glia fibers through the intermediate zone to form six-layer laminar structures (Rakic, 1972; Rakic et al., 2009). Differentiation and synapse formation occur once neurons are properly positioned (Katz and Shatz, 1996; Bystron et al., 2008; Frank and Tsai, 2009). Alterations in any of these processes are involved in pathogenesis of neurodevelopmental disorders such as autism, intellectual disability, and schizophrenia (Fan et al., 2013; Fang et al., 2014; Stoner et al., 2014). TCF4 is present in the VZ/SVZ of the dorsal telencephalon at an early embryonic stage in both humans and mice (de Pontual et al., 2009; Jung et al., 2018). The mouse cortex produces TCF4 protein at the highest level during early embryonic and neonatal development (Chen et al., 2016). Our postnatal immunostaining study shows that TCF4 is upregulated in the mouse cortex at birth, but as mice age, it is downregulated (Figure 5). After birth and through the first 7 to 10 days of postnatal development, cells undergo migration, differentiation, and maturation processes. Therefore, TCF4 is well positioned to influence these critical steps of corticogenesis. TCF4 loss delays neuronal migration, resulting in a thin cortical upper layer (Li et al., 2019). Beyond migration, dendritic and synaptic formation are abnormal in *Tcf4* haploinsufficient mice (Li et al., 2019). These previous and current findings suggest that TCF4 may be an upstream gene of the molecular network regulating migration and maturation processes.

Spatial specificity of axonal projections across different brain regions is important for normal brain development and function (Abelson et al., 2005; Matsuda and Cepko, 2007; Mortazavi et al., 2008), and TCF4 could be positioned to affect such projections. The TCF4 reporter mouse allowed us to visualize projecting axons, as the GFP reporter was free to diffuse throughout the cytoplasmic compartment (Figure 1A). The GFP reporter revealed corticothalamic projections and what appeared to be the corticospinal and corticobulbar tracts (Figure 5A). Because corticothalamic neurons are largely localized in layer 6, and the corticospinal and corticobulbar tracts are largely localized to layer 5 (Chen et al., 2005; Jacobs et al., 2007), our data suggest that TCF4 may be expressed in both layer 5 and 6 projection neurons, although additional experiments will be required to directly confirm this. Several studies demonstrated that TCF4 regulates the laminar pattern and structure of the cortex (Chen et al., 2016; Li et al., 2019), and our findings suggest that TCF4 may also be critical to the development of corticofugal projections. To test this possibility, the consequences of TCF4 loss on axonal projections during embryonic development need to be thoroughly examined.

### Insights Into Genetic Normalization Strategies to Treat TCF4-Related Disorders

TCF4 is a major transcription modulator that differentially controls the expression of hundreds of genes (Forrest et al., 2013; Hill et al., 2017; Xia et al., 2018). Thus, it is wholly impracticable to develop therapeutic tools that adjust the dosage of each impacted gene. Ideally, TCF4-linked disorders can be treated by normalizing TCF4 gene expression levels. A slight upregulation of TCF4 rescues learning and memory phenotypes in adult PTHS mouse model (Kennedy et al., 2016). Studies from similar neurodevelopmental disorders, including Rett and Angelman syndrome, show that reinstatement of affected gene expression can provide therapeutic benefits (Guy et al., 2007; Silva-Santos et al., 2015; Sinnett et al., 2017). These convergent lines of evidence support the idea that TCF4-linked disorders can benefit from normalizing TCF4 levels. Gene therapy using adeno-associated virus (AAV) has been clinically tested as a potential therapeutic intervention for genetic disorders (Deverman et al., 2018; Hudry and Vandenberghe, 2019). In principle, disorders linked to the loss of TCF4 function should be amenable to correction following treatment with viral vectors coding for *TCF4*. Key experimental parameters requiring AAV-mediated gene therapy strategies include distribution of viral vector and the age at time of treatment. TCF4 is distributed in nearly all neurons, astrocytes, and oligodendrocytes in the forebrain at all ages (Figures 6, 7). In contrast, only selective cell types express TCF4 in the striatum, thalamus, midbrain, hindbrain, and cerebellum (Figures 3–5, 8). Optimal design of viral vectors will thus require careful choice of promoter, capsid, and delivery method to promote expression in forebrain neurons over other brain regions. Moreover, microglial cells, medium spiny neurons, ChAT-positive striatal cells, and Purkinje cells lack TCF4 expression (Figures 6–9). Thus, a major challenge for successful therapy is avoiding upregulation of TCF4 in these cell types, as it is unclear how TCF4 expression in these cells will modify the transcriptional machinery.

The other critical parameter that must be considered in treating TCF4-linked disorders is timing of TCF4 expression. Based on the expression profiling of TCF4 (Figure 5; Jung et al., 2018), we predict that earlier interventions will have a larger therapeutic impact on TCF4-linked disorders. After proliferation and maturation, which occur in the prenatal and neonatal periods, there is no need to increase the number of neurons in the brain, except for the hippocampal dentate gyrus. Therefore, after the critical timepoint of neurogenesis and synaptogenesis, the brain undergoes limited plastic changes (Bystron et al., 2008; Budday et al., 2015). Late onset therapies are unlikely to exert as dramatic a phenotypic improvement compared to early intervention, yet partial improvement of some phenotypes in adults or prevention of disease progression would be significant achievements. Our novel TCF4 conditional mouse model allows us to reinstate wildtype *Tcf4* under its own promoter and regulatory elements (Figure 1A). Using this powerful tool, future experiments must be performed to determine the latest age by which normalizing TCF4 expression can improve or even rescue PTHS-associated phenotypes.

## Data Availability Statement

The raw data supporting the conclusions of this article will be made available by the authors, without undue reservation.

## Ethics Statement

The animal study was reviewed and approved by the Institutional Animal Care and Use Committee at the University of North Carolina at Chapel Hill.

## Author Contributions

HK designed the experiments with guidance from BP, performed the experiments, and analyzed the data. NB and NO performed *in situ* hybridizations and provided histological assistance. HK and BP wrote the manuscript. All authors contributed scientific insights and provided critical readings of the manuscript.

## Conflict of Interest

The authors declare that the research was conducted in the absence of any commercial or financial relationships that could be construed as a potential conflict of interest.

## References

[B1] Abelson J. F. Kwan K. Y. O’Roak B. J. Baek D. Y. Stillman A. A. Morgan T. M. (2005). Sequence variants in SLITRK1 are associated with Tourette’s syndrome. *Science* 310 317–320. 10.1126/science.1116502 16224024

[B2] Altman J. (1969). Autoradiographic and histological studies of postnatal neurogenesis. IV. Cell proliferation and migration in the anterior forebrain, with special reference to persisting neurogenesis in the olfactory bulb. *J. Comp. Neurol.* 137 433–457. 10.1002/cne.901370404 5361244

[B3] Amiel J. Rio M. de Pontual L. Redon R. Malan V. Boddaert N. (2007). Mutations in TCF4, encoding a class I basic helix-loop-helix transcription factor, are responsible for Pitt-Hopkins syndrome, a severe epileptic encephalopathy associated with autonomic dysfunction. *Am. J. Hum. Genet.* 80 988–993. 10.1086/515582 17436254 PMC1852736

[B4] Basmanav F. B. Forstner A. J. Fier H. Herms S. Meier S. Degenhardt F. (2015). Investigation of the role of TCF4 rare sequence variants in schizophrenia. *Am. J. Med. Genet. B Neuropsychiatr. Genet.* 168B 354–362. 10.1002/ajmg.b.32318 26010163

[B5] Bedeschi M. F. Marangi G. Calvello M. R. Ricciardi S. Leone F. P. C. Baccarin M. (2017). Impairment of different protein domains causes variable clinical presentation within Pitt-Hopkins syndrome and suggests intragenic molecular syndromology of TCF4. *Eur. J. Med. Genet.* 60 565–571. 10.1016/j.ejmg.2017.08.004 28807867

[B6] Bhat R. V. Axt K. J. Fosnaugh J. S. Smith K. J. Johnson K. A. Hill D. E. (1996). Expression of the APC tumor suppressor protein in oligodendroglia. *Glia* 17 169–174. 8776583 10.1002/(SICI)1098-1136(199606)17:2<169::AID-GLIA8>3.0.CO;2-Y

[B7] Bolduc M. E. du Plessis A. J. Sullivan N. Guizard N. Zhang X. Robertson R. L. (2012). Regional cerebellar volumes predict functional outcome in children with cerebellar malformations. *Cerebellum* 11 531–542. 10.1007/s12311-011-0312-z 21901523

[B8] Budday S. Steinmann P. Kuhl E. (2015). Physical biology of human brain development. *Front. Cell. Neurosci.* 9:257. 10.3389/fncel.2015.00257 26217183 PMC4495345

[B9] Bystron I. Blakemore C. Rakic P. (2008). Development of the human cerebral cortex: Boulder Committee revisited. *Nat. Rev. Neurosci.* 9 110–122. 10.1038/nrn2252 18209730

[B10] Chen B. Schaevitz L. R. McConnell S. K. (2005). Fezl regulates the differentiation and axon targeting of layer 5 subcortical projection neurons in cerebral cortex. *Proc. Natl. Acad. Sci. U.S.A.* 102 17184–17189. 10.1073/pnas.0508732102 16284245 PMC1282569

[B11] Chen T. Wu Q. Zhang Y. Lu T. Yue W. Zhang D. (2016). Tcf4 Controls Neuronal Migration of the Cerebral Cortex through Regulation of Bmp7. *Front. Mol. Neurosci.* 9:94. 10.3389/fnmol.2016.00094 27752241 PMC5046712

[B12] de Pontual L. Mathieu Y. Golzio C. Rio M. Malan V. Boddaert N. (2009). Mutational, functional, and expression studies of the TCF4 gene in Pitt-Hopkins syndrome. *Hum. Mutat.* 30 669–676. 10.1002/humu.20935 19235238

[B13] DeFelipe J. (1993). Neocortical neuronal diversity: chemical heterogeneity revealed by colocalization studies of classic neurotransmitters, neuropeptides, calcium-binding proteins, and cell surface molecules. *Cereb. Cortex* 3 273–289. 10.1093/cercor/3.4.273 8104567

[B14] Deverman B. E. Ravina B. M. Bankiewicz K. S. Paul S. M. Sah D. W. Y. (2018). Gene therapy for neurological disorders: progress and prospects. *Nat. Rev. Drug Discov.* 17:767. 10.1038/nrd.2018.158 30206384

[B15] Dupret D. O’Neill J. Pleydell-Bouverie B. Csicsvari J. (2010). The reorganization and reactivation of hippocampal maps predict spatial memory performance. *Nat. Neurosci.* 13 995–1002. 10.1038/nn.2599 20639874 PMC2923061

[B16] Fan Y. Abrahamsen G. Mills R. Calderon C. C. Tee J. Y. Leyton L. (2013). Focal adhesion dynamics are altered in schizophrenia. *Biol. Psychiatry* 74 418–426. 10.1016/j.biopsych.2013.01.020 23482246

[B17] Fang W. Q. Chen W. W. Jiang L. Liu K. Yung W. H. Fu A. K. Y. (2014). Overproduction of upper-layer neurons in the neocortex leads to autism-like features in mice. *Cell Rep.* 9 1635–1643. 10.1016/j.celrep.2014.11.003 25466248

[B18] Forrest M. P. Hill M. J. Kavanagh D. H. Tansey K. E. Waite A. J. Blake D. J. (2018). The psychiatric risk gene transcription factor 4 (TCF4) regulates neurodevelopmental pathways associated with schizophrenia, autism, and intellectual disability. *Schizophr. Bull.* 44 1100–1110. 10.1093/schbul/sbx164 29228394 PMC6101561

[B19] Forrest M. P. Waite A. J. Martin-Rendon E. Blake D. J. (2013). Knockdown of human TCF4 affects multiple signaling pathways involved in cell survival, epithelial to mesenchymal transition and neuronal differentiation. *PLoS One* 8:e73169. 10.1371/journal.pone.0073169 24058414 PMC3751932

[B20] Frank C. L. Tsai L. H. (2009). Alternative functions of core cell cycle regulators in neuronal migration, neuronal maturation, and synaptic plasticity. *Neuron* 62 312–326. 10.1016/j.neuron.2009.03.029 19447088 PMC2757047

[B21] Fu H. Cai J. Clevers H. Fast E. Gray S. Greenberg R. (2009). A genome-wide screen for spatially restricted expression patterns identifies transcription factors that regulate glial development. *J. Neurosci.* 29 11399–11408. 10.1523/JNEUROSCI.0160-09.2009 19741146 PMC2775518

[B22] Gittis A. H. Nelson A. B. Thwin M. T. Palop J. J. Kreitzer A. C. (2010). Distinct roles of GABAergic interneurons in the regulation of striatal output pathways. *J. Neurosci.* 30 2223–2234. 10.1523/JNEUROSCI.4870-09.2010 20147549 PMC2836801

[B23] Goebbels S. Bormuth I. Bode U. Hermanson O. Schwab M. H. Nave K. A. (2006). Genetic targeting of principal neurons in neocortex and hippocampus of NEX-Cre mice. *Genesis* 44 611–621. 10.1002/dvg.20256 17146780

[B24] Gonchar Y. Burkhalter A. (1997). Three distinct families of GABAergic neurons in rat visual cortex. *Cereb. Cortex* 7 347–358. 10.1093/cercor/7.4.347 9177765

[B25] Goodspeed K. Newsom C. Morris M. A. Powell C. Evans P. Golla S. (2018). Pitt-Hopkins syndrome: a review of current literature, clinical approach, and 23-patient case series. *J. Child Neurol.* 33 233–244. 10.1177/0883073817750490 29318938 PMC5922265

[B26] Guy J. Gan J. Selfridge J. Cobb S. Bird A. (2007). Reversal of neurological defects in a mouse model of Rett syndrome. *Science* 315 1143–1147. 10.1126/science.1138389 17289941 PMC7610836

[B27] Hill M. J. Killick R. Navarrete K. Maruszak A. McLaughlin G. M. Williams B. P. (2017). Knockdown of the schizophrenia susceptibility gene TCF4 alters gene expression and proliferation of progenitor cells from the developing human neocortex. *J. Psychiatry Neurosci.* 42 181–188. 27689884 10.1503/jpn.160073 PMC5403663

[B28] Hudry E. Vandenberghe L. H. (2019). Therapeutic AAV gene transfer to the nervous system: a clinical reality. *Neuron* 102:263. 10.1016/j.neuron.2019.03.020 30946822

[B29] Jacobs E. C. Campagnoni C. Kampf K. Reyes S. D. Kalra V. Handley V. (2007). Visualization of corticofugal projections during early cortical development in a tau-GFP-transgenic mouse. *Eur. J. Neurosci.* 25 17–30. 10.1111/j.1460-9568.2006.05258.x 17241263

[B30] Jung M. Haberle B. M. Tschaikowsky T. Wittmann M. T. Balta E. A. Stadler V. C. (2018). Analysis of the expression pattern of the schizophrenia-risk and intellectual disability gene TCF4 in the developing and adult brain suggests a role in development and plasticity of cortical and hippocampal neurons. *Mol. Autism* 9:20. 10.1186/s13229-018-0200-1 29588831 PMC5863811

[B31] Kamentsky L. Jones T. R. Fraser A. Bray M. A. Logan D. J. Madden K. L. (2011). Improved structure, function and compatibility for CellProfiler: modular high-throughput image analysis software. *Bioinformatics* 27 1179–1180. 10.1093/bioinformatics/btr095 21349861 PMC3072555

[B32] Katz L. C. Shatz C. J. (1996). Synaptic activity and the construction of cortical circuits. *Science* 274 1133–1138. 10.1126/science.274.5290.1133 8895456

[B33] Kennedy A. J. Rahn E. J. Paulukaitis B. S. Savell K. E. Kordasiewicz H. B. Wang J. (2016). Tcf4 regulates synaptic plasticity, DNA methylation, and memory function. *Cell Rep.* 16 2666–2685. 10.1016/j.celrep.2016.08.004 27568567 PMC5710002

[B34] Kharbanda M. Kannike K. Lampe A. Berg J. Timmusk T. Sepp M. (2016). Partial deletion of TCF4 in three generation family with non-syndromic intellectual disability, without features of Pitt-Hopkins syndrome. *Eur. J. Med. Genet.* 59 310–314. 10.1016/j.ejmg.2016.04.003 27132474

[B35] Koos T. Tepper J. M. (1999). Inhibitory control of neostriatal projection neurons by GABAergic interneurons. *Nat. Neurosci.* 2 467–472. 10.1038/8138 10321252

[B36] Kreitzer A. C. Malenka R. C. (2008). Striatal plasticity and basal ganglia circuit function. *Neuron* 60 543–554. 10.1016/j.neuron.2008.11.005 19038213 PMC2724179

[B37] Lee S. Hjerling-Leffler J. Zagha E. Fishell G. Rudy B. (2010). The largest group of superficial neocortical GABAergic interneurons expresses ionotropic serotonin receptors. *J. Neurosci.* 30 16796–16808. 10.1523/JNEUROSCI.1869-10.2010 21159951 PMC3025500

[B38] Li H. Zhu Y. Morozov Y. M. Chen X. Page S. C. Rannals M. D. (2019). Disruption of TCF4 regulatory networks leads to abnormal cortical development and mental disabilities. *Mol. Psychiatry* 24 1235–1246. 10.1038/s41380-019-0353-0 30705426 PMC11019556

[B39] Ma C. Gu C. Huo Y. Li X. Luo X. J. (2018). The integrated landscape of causal genes and pathways in schizophrenia. *Transl. Psychiatry* 8:67. 10.1038/s41398-018-0114-x 29540662 PMC5851982

[B40] Maduro V. Pusey B. N. Cherukuri P. F. Atkins P. du Souich C. Rupps R. (2016). Complex translocation disrupting TCF4 and altering TCF4 isoform expression segregates as mild autosomal dominant intellectual disability. *Orphanet. J. Rare Dis.* 11:62. 10.1186/s13023-016-0439-6 27179618 PMC4868023

[B41] Maia T. V. Frank M. J. (2011). From reinforcement learning models to psychiatric and neurological disorders. *Nat. Neurosci.* 14 154–162. 10.1038/nn.2723 21270784 PMC4408000

[B42] Markram H. Toledo-Rodriguez M. Wang Y. Gupta A. Silberberg G. Wu C. (2004). Interneurons of the neocortical inhibitory system. *Nat. Rev. Neurosci.* 5 793–807. 10.1038/nrn1519 15378039

[B43] Mary L. Piton A. Schaefer E. Mattioli F. Nourisson E. Feger C. (2018). Disease-causing variants in TCF4 are a frequent cause of intellectual disability: lessons from large-scale sequencing approaches in diagnosis. *Eur. J. Hum. Genet.* 26 996–1006. 10.1038/s41431-018-0096-4 29695756 PMC6018712

[B44] Massari M. E. Murre C. (2000). Helix-loop-helix proteins: regulators of transcription in eucaryotic organisms. *Mol. Cell. Biol.* 20 429–440. 10.1128/mcb.20.2.429-440.2000 10611221 PMC85097

[B45] Matsuda T. Cepko C. L. (2007). Controlled expression of transgenes introduced by in vivo electroporation. *Proc. Natl. Acad. Sci. U.S.A.* 104 1027–1032. 10.1073/pnas.0610155104 17209010 PMC1764220

[B46] Miller E. K. (2000). The prefrontal cortex and cognitive control. *Nat. Rev. Neurosci.* 1 59–65. 10.1038/35036228 11252769

[B47] Mortazavi A. Williams B. A. McCue K. Schaeffer L. Wold B. (2008). Mapping and quantifying mammalian transcriptomes by RNA-Seq. *Nat. Methods* 5 621–628. 10.1038/nmeth.1226 18516045 PMC13303166

[B48] Munoz-Manchado A. B. Bengtsson Gonzales C. Zeisel A. Munguba H. Bekkouche B. Skene N. G. (2018). Diversity of interneurons in the dorsal striatum revealed by single-cell RNA Sequencing and PatchSeq. *Cell Rep.* 24 2179–2190.e7. 10.1016/j.celrep.2018.07.053 30134177 PMC6117871

[B49] Murre C. Bain G. van Dijk M. A. Engel I. Furnari B. A. Massari M. E. (1994). Structure and function of helix-loop-helix proteins. *Biochim. Biophys. Acta* 1218 129–135. 10.1016/0167-4781(94)90001-9 8018712

[B50] Peippo M. M. Simola K. O. Valanne L. K. Larsen A. T. Kahkonen M. Auranen M. P. (2006). Pitt-Hopkins syndrome in two patients and further definition of the phenotype. *Clin. Dysmorphol.* 15 47–54. 10.1097/01.mcd.0000184973.14775.32 16531728

[B51] Pelkey K. A. Chittajallu R. Craig M. T. Tricoire L. Wester J. C. McBain C. J. (2017). Hippocampal GABAergic Inhibitory Interneurons. *Physiol. Rev.* 97 1619–1747. 10.1152/physrev.00007.2017 28954853 PMC6151493

[B52] Phan B. N. Bohlen J. F. Davis B. A. Ye Z. Chen H. Y. Mayfield B. (2020). A myelin-related transcriptomic profile is shared by Pitt-Hopkins syndrome models and human autism spectrum disorder. *Nat. Neurosci.* 23 375–385. 10.1038/s41593-019-0578-x 32015540 PMC7065955

[B53] Pickard B. S. Malloy M. P. Clark L. Lehellard S. Ewald H. L. Mors O. (2005). Candidate psychiatric illness genes identified in patients with pericentric inversions of chromosome 18. *Psychiatr. Genet.* 15 37–44. 10.1097/00041444-200503000-00007 15722956

[B54] Rakic P. (1972). Mode of cell migration to the superficial layers of fetal monkey neocortex. *J. Comp. Neurol.* 145 61–83. 10.1002/cne.901450105 4624784

[B55] Rakic P. Ayoub A. E. Breunig J. J. Dominguez M. H. (2009). Decision by division: making cortical maps. *Trends Neurosci.* 32 291–301. 10.1016/j.tins.2009.01.007 19380167 PMC3601545

[B56] Rannals M. D. Hamersky G. R. Page S. C. Campbell M. N. Briley A. Gallo R. A. (2016). Psychiatric risk gene transcription factor 4 regulates intrinsic excitability of prefrontal neurons via repression of SCN10a and KCNQ1. *Neuron* 90 43–55. 10.1016/j.neuron.2016.02.021 26971948 PMC4824652

[B57] Rapanelli M. Frick L. R. Pittenger C. (2017). The role of interneurons in autism and Tourette syndrome. *Trends Neurosci.* 40 397–407. 10.1016/j.tins.2017.05.004 28578790 PMC5528854

[B58] Rogers T. D. McKimm E. Dickson P. E. Goldowitz D. Blaha C. D. Mittleman G. (2013). Is autism a disease of the cerebellum? An integration of clinical and pre-clinical research. *Front. Syst. Neurosci.* 7:15. 10.3389/fnsys.2013.00015 23717269 PMC3650713

[B59] Rubin R. D. Watson P. D. Duff M. C. Cohen N. J. (2014). The role of the hippocampus in flexible cognition and social behavior. *Front. Hum. Neurosci.* 8:742. 10.3389/fnhum.2014.00742 25324753 PMC4179699

[B60] Rudy B. Fishell G. Lee S. Hjerling-Leffler J. (2011). Three groups of interneurons account for nearly 100% of neocortical GABAergic neurons. *Dev. Neurobiol.* 71 45–61. 10.1002/dneu.20853 21154909 PMC3556905

[B61] Schmahmann J. D. Caplan D. (2006). Cognition, emotion and the cerebellum. *Brain* 129(Pt 2), 290–292. 10.1093/brain/awh729 16434422

[B62] Sepp M. Kannike K. Eesmaa A. Urb M. Timmusk T. (2011). Functional diversity of human basic helix-loop-helix transcription factor TCF4 isoforms generated by alternative 5′ exon usage and splicing. *PLoS One* 6:e22138. 10.1371/journal.pone.0022138 21789225 PMC3137626

[B63] Sepp M. Pruunsild P. Timmusk T. (2012). Pitt-Hopkins syndrome-associated mutations in TCF4 lead to variable impairment of the transcription factor function ranging from hypomorphic to dominant-negative effects. *Hum. Mol. Genet.* 21 2873–2888. 10.1093/hmg/dds112 22460224

[B64] Silva-Santos S. van Woerden G. M. Bruinsma C. F. Mientjes E. Jolfaei M. A. Distel B. (2015). Ube3a reinstatement identifies distinct developmental windows in a murine Angelman syndrome model. *J. Clin. Invest.* 125 2069–2076. 10.1172/JCI80554 25866966 PMC4463212

[B65] Sinnett S. E. Hector R. D. Gadalla K. K. E. Heindel C. Chen D. Zaric V. (2017). Improved MECP2 gene therapy extends the survival of MeCP2-null mice without apparent toxicity after intracisternal delivery. *Mol. Ther. Methods Clin. Dev.* 5 106–115. 10.1016/j.omtm.2017.04.006 28497072 PMC5424572

[B66] Skene N. G. Bryois J. Bakken T. E. Breen G. Crowley J. J. Gaspar H. A. (2018). Genetic identification of brain cell types underlying schizophrenia. *Nat. Genet.* 50 825–833. 10.1038/s41588-018-0129-5 29785013 PMC6477180

[B67] Steinlin M. (2008). Cerebellar disorders in childhood: cognitive problems. *Cerebellum* 7 607–610. 10.1007/s12311-008-0083-3 19057977

[B68] Stoner R. Chow M. L. Boyle M. P. Sunkin S. M. Mouton P. R. Roy S. (2014). Patches of disorganization in the neocortex of children with autism. *N. Engl. J. Med.* 370 1209–1219. 10.1056/NEJMoa1307491 24670167 PMC4499461

[B69] Straub C. Saulnier J. L. Begue A. Feng D. D. Huang K. W. Sabatini B. L. (2016). Principles of synaptic organization of GABAergic interneurons in the striatum. *Neuron* 92 84–92. 10.1016/j.neuron.2016.09.007 27710792 PMC5074692

[B70] Taniguchi H. He M. Wu P. Kim S. Paik R. Sugino K. (2011). A resource of Cre driver lines for genetic targeting of GABAergic neurons in cerebral cortex. *Neuron* 71 995–1013. 10.1016/j.neuron.2011.07.026 21943598 PMC3779648

[B71] Thaxton C. Kloth A. D. Clark E. P. Moy S. S. Chitwood R. A. Philpot B. D. (2018). Common pathophysiology in multiple mouse models of pitt-hopkins syndrome. *J. Neurosci.* 38 918–936. 10.1523/JNEUROSCI.1305-17.2017 29222403 PMC5783968

[B72] Wang F. Flanagan J. Su N. Wang L. C. Bui S. Nielson A. (2012). RNAscope: a novel in situ RNA analysis platform for formalin-fixed, paraffin-embedded tissues. *J. Mol. Diagn.* 14 22–29. 10.1016/j.jmoldx.2011.08.002 22166544 PMC3338343

[B73] Wang Y. Lu Z. Zhang Y. Cai Y. Yun D. Tang T. (2020). Transcription Factor 4 safeguards hippocampal dentate gyrus development by regulating neural progenitor migration. *Cereb. Cortex* 30 3102–3115. 10.1093/cercor/bhz297 31845732

[B74] Whalen S. Heron D. Gaillon T. Moldovan O. Rossi M. Devillard F. (2012). Novel comprehensive diagnostic strategy in Pitt-Hopkins syndrome: clinical score and further delineation of the TCF4 mutational spectrum. *Hum. Mutat.* 33 64–72. 10.1002/humu.21639 22045651

[B75] Xia H. Jahr F. M. Kim N. K. Xie L. Shabalin A. A. Bryois J. (2018). Building a schizophrenia genetic network: transcription factor 4 regulates genes involved in neuronal development and schizophrenia risk. *Hum. Mol. Genet.* 27 3246–3256. 10.1093/hmg/ddy222 29905862 PMC6354221

[B76] Zollino M. Zweier C. Van Balkom I. D. Sweetser D. A. Alaimo J. Bijlsma E. K. (2019). Diagnosis and management in Pitt-Hopkins syndrome: first international consensus statement. *Clin. Genet.* 95 462–478. 10.1111/cge.13506 30677142

[B77] Zweier C. Peippo M. M. Hoyer J. Sousa S. Bottani A. Clayton-Smith J. (2007). Haploinsufficiency of TCF4 causes syndromal mental retardation with intermittent hyperventilation (Pitt-Hopkins syndrome). *Am. J. Hum. Genet.* 80 994–1001. 10.1086/515583 17436255 PMC1852727

[B78] Zweier C. Sticht H. Bijlsma E. K. Clayton-Smith J. Boonen S. E. Fryer A. (2008). Further delineation of Pitt-Hopkins syndrome: phenotypic and genotypic description of 16 novel patients. *J. Med. Genet.* 45 738–744. 10.1136/jmg.2008.060129 18728071

